# Female Pattern Hair Loss

**DOI:** 10.5812/ijem.9860

**Published:** 2013-10-21

**Authors:** Ingrid Herskovitz, Antonella Tosti

**Affiliations:** 1 Department of Dermatology and Cutaneous Surgery, University of Miami, Miami, USA

**Keywords:** Polycystic Ovary Syndrome, Minoxidil, Female, Alopecia, Therapy, Alopecia, physiopathology, Androgen antagonist, Therapeutic Use

## Abstract

**Context:**

Female pattern hair loss (FPHL) also known as female androgenetic alopecia is a common condition afflicting millions of women that can be cosmetically disrupting. Prompt diagnosis and treatment are essential for obtaining optimal outcome.

This review addresses the clinical presentation of female pattern hair loss, its differential diagnosis and treatment modalities.

**Evidence Acquisition:**

A) Diffuse thinning of the crown region with preservation of the frontal hairline (Ludwig’s type)

B) The “Christmas tree pattern” where the thinning is wider in the frontal scalp giving the alopecic area a triangular shaped figure resembling a christmas tree.

C) Thinning associated with bitemporal recession (Hamilton type).

Generally, FPHL is not associated with elevated androgens.

Less commonly females with FPHL may have other skin or general signs of hyperandrogenism such as hirsutism, acne, irregular menses, infertility, galactorrhea and insulin resistance. The most common endocrinological abnormality associated with FPHL is polycystic ovarian syndrome (PCOS).

**Results:**

The most important diseases to consider in the differential diagnosis of FPHL include Chronic Telogen Effluvium (CTE), Permanent Alopecia after Chemotherapy (PAC), Alopecia Areata Incognito (AAI) and Frontal Fibrosing Alopecia (FFA). This review describes criteria for distinguishing these conditions from FPHL.

**Conclusions:**

The only approved treatment for FPHL, which is 2% topical Minoxidil, should be applied at the dosage of 1ml twice day for a minimum period of 12 months. This review will discuss off-label alternative modalities of treatment including 5-alfa reductase inhibitors, antiandrogens, estrogens, prostaglandin analogs, lasers, light treatments and hair transplantation.

## 1. Female Pattern Hair Loss 

FPHL has been defined as nonscarring progressive miniaturization of the hair follicle, usually with characteristic pattern distribution that occurs in genetically predisposed women (1).

Female Pattern Hair Loss (FPHL) is a frequent diagnosis in the medical practice. The intention of this review is to clarify the guidelines for diagnosis and treatment and address the clinical presentation of female pattern hair loss, its differential diagnosis and treatment modalities.

The review search included Medline, Google Scholar and LILACS between 1977 and 2012. The keywords utilized were: female pattern hair loss, hair growth and development, alopecia/physiopathology, alopecia/chemically induced, polycystic ovary syndrome/diagnosis, minoxidil/administration and dosage, alopecia/therapy, androgen antagonists/therapeutic use.

## 2. Epidemiology

Female pattern hair loss is a frequent condition. There are over 21 million women affected by FPHL in the U.S.A (2). This disease is important because of the intense emotional distress it causes by disrupting self image (3).

There are reported incidences of 12% in females around 30 years old and of 30-40% in the female population between 60 and 69 years old (4, 5). This condition usually manifests after puberty with variable clinical severity and rate of progression but can manifest at any age. The earlier it presents the more intense the clinical picture tends to be.

Female pattern hair loss is also termed female androgenetic alopecia because its possible association with altered androgen metabolism and familial occurence.

## 3. Etiopathogenesis

The hair follicles are constantly cycling between growth and rest. The growth phase will determine the length of the hair.

Most scalp hairs (85-90%) are in the anagen phase, which is the growth stage of the hair follicle cycle and lasts for approximately 2-6 years. Ten to fifteen percent of hairs are in the telogen phase which is the resting phase of the hair follicle cycle and lasts for about 3 months. The hair shaft sheds at the end of the telogen phase. Hair may vary in shaft diameter and length: vellus hairs are depigmented usually non-medulated, thinner and shorter than terminal hairs, which are pigmented and have a medulla. While they both undergo the entire hair cycle, the cycle is shorter for vellus hair. 

In FPHL there is progressive hair follicle miniaturization and conversion of terminal follicles into vellus-like follicles. These vellus-like follicles have a shortened hair cycle because their anagen phase is reduced and produce hair shafts that are short and fine. Unlike in men, the miniaturization is not uniform and intense, therefore, except for very rare cases, there are no complete areas of baldness (4).

In some women with FPHL there is evidence of altered metabolism of androgens but excessive androgen production is not present in all cases. Rather, since serum testosterone is normal in most patients (6-8) the term Female Pattern Hair Loss is preferred over “Female Androgenetic Alopecia”. An increased sensitivity of the hair follicle to normal androgen levels can explain the onset of the disease in patients without hyperandrogenism. To further complicate the problem there are individuals with androgen insensitivity syndrome or alpha reductase deficiency, who present with patterned scalp alopecia (9). This indicates that FPHL, differently from male pattern hair loss, may develop even in the absence of androgens. A recent questionnaire based study even showed that androgen treatment can improve FPHL in some women. The study monitored for 1 year pre and postmenopausal patients for the effects of subcutaneous testosterone implants on the incidence of breast cancer. Hair thinning was assessed and 63% of patient who complained of hair thinning reported hair regrowth with treatment (10).

When androgens levels are elevated the role of these hormones is quite clear although it is noteworthy that hyperandrogenism by itself does not necessarely cause FPHL (11). The mechanism by which androgens cause hair loss has been linked to increased production of cytokines, which induce the hair to enter the telogen phase and the dermal papilla to become senescent (12, 13).

The genetic inheritance of FPHL is still unclear. FPHL is possibly a multigenic disease, but the causative genes are not established. The polymorphism of one of the two major susceptibility genes for male pattern hairloss, the androgen receptor gene EBA2R on the X chromosome, has been recently associated with early onset FPHL (14) . The role of aromates genes CYP19A1) (15) has been reported but not confirmed in a more recent study (16). In other studies there were no associations between steroid 5-alpha-reductase isoforms genes or sex steroid hormone receptors and FPHL (17). Neither there was association with melanocortin 4 receptor gene (18).

FPHL is often precipitated and exacerbated by conditions that cause telogen effluvium, such as medications, acute stressors, weight loss, partum and hormonal therapies with proandrogenic effects like norethisterone, levornogestrel and tibolon. 

The association with other skin signs of hyperandrogenism such as acne or hirsutism is an indication for extensive interdisciplinary evaluation (1).

## 4. Clinical Features

The female pattern hair loss patient usually complains of slowly progressive hair thinning (1) that may or may not be associated with increased shedding. The affected areas usually involve the vertex and upper parietal scalp and sometimes also frontoparietal areas of the head. Unike in men the frontal hair line is typically preserved and the hair miniaturization is not as severe.

**Figure 1.**
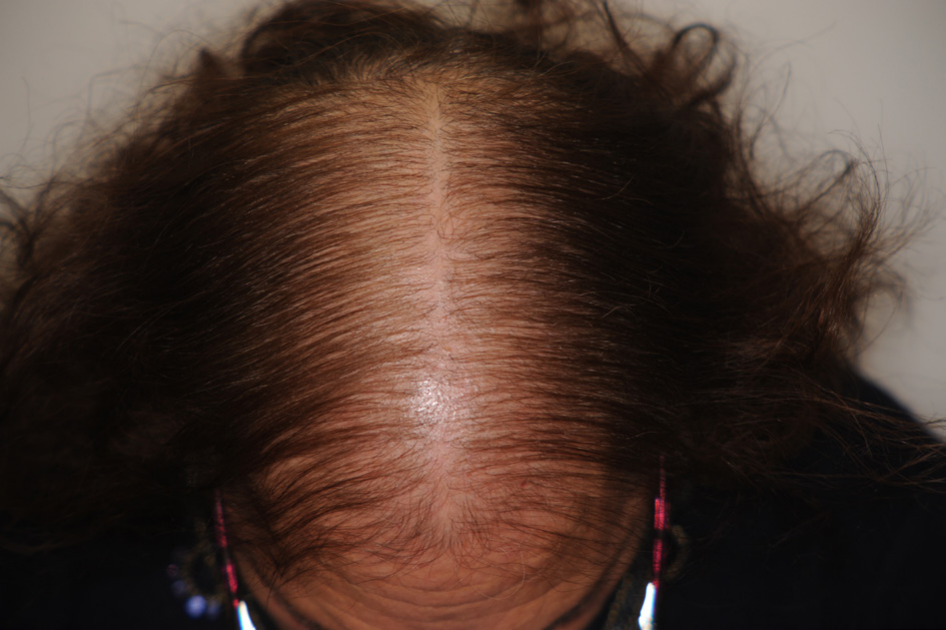
FPHL: Ludwig Type Severity: Ludwig 2/ Sinclair 4

Three different patterns of hair loss can be observed: 

1. Diffuse thinning of the crown region with preservation of the frontal hairline (Ludwig’s type) (Figure 1). The severity of this pattern can be evaluated using the 3-point Ludwig ( 19 ) scale or the 5-point Sinclair scale. 

2. Frontal midline recession/breach with thinning and widening of the central part of the scalp without diffuse hair loss, best known as “Christmas tree pattern” as described by Olsen (20). This pattern also involves the superior part of the scalp but the thinning is wider in the frontal scalp giving the alopecic area a triangular shaped figure resembling a christmas tree.

3. Thinning associated with bitemporal recession (Hamilton type). This presentation has the same classical distribution of male pattern baldness: thinning evident in the lateral-frontal part of the superior scalp and vertex.

According with a study, women with Ludwig’s pattern may develop an Hamilton’s pattern after menopause (21).In some women, however, hair thinning is more diffuse and involves the parietal and occipital areas of the scalp, with a pattern of diffuse alopecia. These cases might impose an obstacle when in search of donor areas for hair transplantation (1).

## 5. Diagnosis

The diagnosis is mainly clinical and biopsy is usually not necessary. Dermoscopy is useful to detect early FPHL and to distinguish FPHL from other hair disorders that can cause hair thinning (22).

The hair pull test, which is a maneuver performed by the examiner that gently pulls tufts of hairs along the scalp, is usually positive in the affected scalp as miniaturization causes shortening of the hair cycle with increased telogen shedding. When positive in all scalp areas it indicates associated telogen effluvium. Dermoscopic examination of the scalp correlates with the clinical classifications of FPHL, revealing variability in the hair shaft diameter that affects at least 20% of hairs (23) and increased number of vellus hairs, parameters which are linked to follicle miniaturization (24).

Patients with FPHL may have other skin or general signs of hyperandrogenism such as hirsutism, acne, irregular menses, infertility, galactorrhea and insulin resistance, but most do not. The most common endocrinological abnormality associated with FPHL is polycystic ovarian syndrome (PCOS). Hyperandrogenism is often a common feature between the two conditions and in both, the manifestation of this hyperandrogenism may not correlate with the circulating androgen levels because total circulating testosterone is mostly bound to albumin and sex-hormone binding globulin. But even then, both conditions can be treated with anti-androgens, androgen receptor blockers and enzyme inhibitors to avoid the effects of the androgens in the target organs. Another important association with FPHL is metabolic syndrome because of increased cardiovascular risks. One study with 77 female subjects found 48.6% of the women with FPHL to also have metabolic syndrome (25) and a more recent study conducted in 1701 women in Korea, proved positive statistical association between FPHL and metabolic syndrome (26). Possible mechanisms to explain the association between these conditions are the presence of 5 alpha-reductase and DHT receptors in the vessels. 

Patients must also be investigated for systemic and newly diagnosed illnessess within the past year before the signs of alopecia manifested, as well as about significant weight loss, eating habits and medications that can cause hair loss or increase androgen levels (1).

## 6. Laboratory Tests

It is important to evaluate thyroid function, ferritin and vitamin D level to exclude factors that can increase hair shedding and aggravate the disease.

Patients with a history of irregular menses, elevated body mass index or skin signs of hyperandrogenism should be referred to endocrinologists for possible PCOS. Laboratory tests utilized to evaluate androgen state are reported in Table 1.

**Table 1.** Laboratory Tests to Exclude Androgen Excess

**5 alpha dehydrotestosterone**
**17 beta hydroxyprogesterone**
**Androstenedione**
**Dehydroepiandrosterone**
**Total testosterone/SHBG ratio**
**Sex hormone-binding globulin**

## 7. Differential Diagnosis

In our experience the most important diseases to consider in the differential diagnosis of FPHL include Chronic Telogen Effluvium (CTE), Permanent Alopecia after Chemotherapy (PAC), Alopecia Areata Incognito (AAI) and Frontal Fibrosing Alopecia (FFA). 

**Figure 2.**
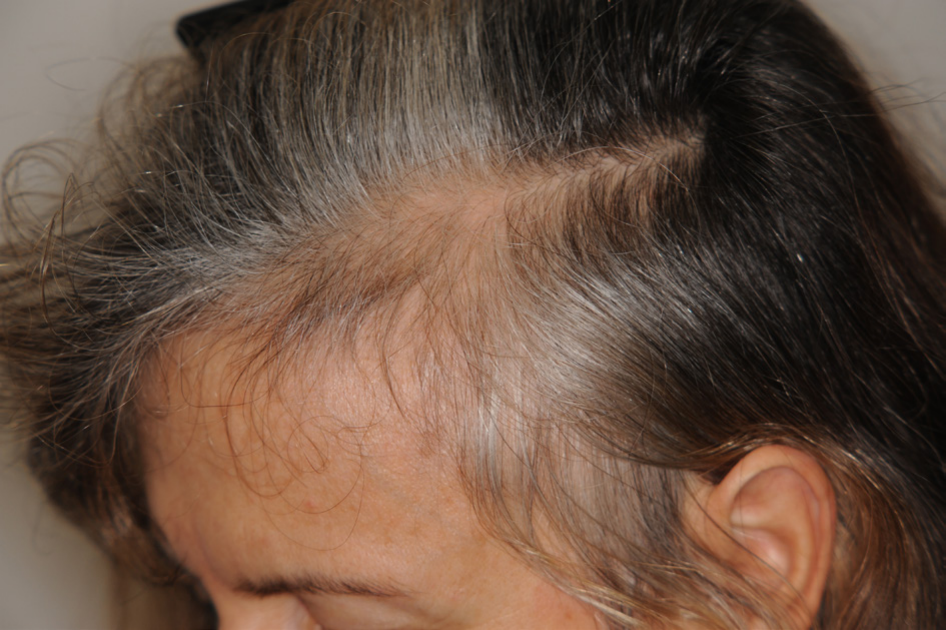
CTE: Note Typical Temporal Recession. The hair density is otherwise normal

Chronic Telogen Effluvium (CTE) is a primary idiopathic disease that most commonly affects middle aged women who complain of increased hair shedding, with bitemporal recession (Figure 2) ( 27 ). Hair thinning is not a feature of CTE and patients usually have a very good hair density despite complaining of reduction of their hair volume. There is no miniaturization ( 27 , 28 ) and some patients may complain of trichodynia. The cause maybe multifactorial and difficult to establish. Usually there is not a triggering factor, as in acute telogen effluvium. 

CTE can be differentiated from early FPHL by dermoscopy and histology (22, 23, 27) 

Some authors believe CTE tends to spontaneously improve over a decade of fluctuation of the disease, cycling every two years, according to one experimental study (29). This condition does not tend to progress to baldness.

Permanent alopecia after chemotherapy (PAC) is defined as incomplete hair regrowth after chemotherapy, possibly due to hair follicle stem cell distruction. The cause remains unknown. The most commonly implicated agents are busulphan (Bu)/cyclophosphamides (Cy)-drugs used in conditioning treatments for bone marrow transplantation (BMT) and taxanes (docetaxel, paclitaxel (30, 31). The frequency of PCIA varies according to agent and dose utilized for chemotherapy.

Patients have moderate to very severe hair thinning, with short miniaturized hairs. Hair thinning may be more evident on androgen-dependent scalp regions (30, 32). In one study, patients complained the hair would not grow longer than 10cm and in some cases, the texture of the regrown hair would be different. The disease is irreversible and so far there has been no effective treatment (31). Alopecia Areata Incognito is a variant of Alopecia Areata (33, 34) characterized by severe acute thinning and hair shedding of telogen roots in different stages of maturation (33) Patients may develop classical patches of alopecia areata in the follow up. Diagnosis requires histopathological examination, but can be suggested by dermoscopy, which shows yellow dots and short regrowing hairs (33, 35). The prognosis is usually favorable with rapid response to steroid treatment (33).

**Figure 3.**
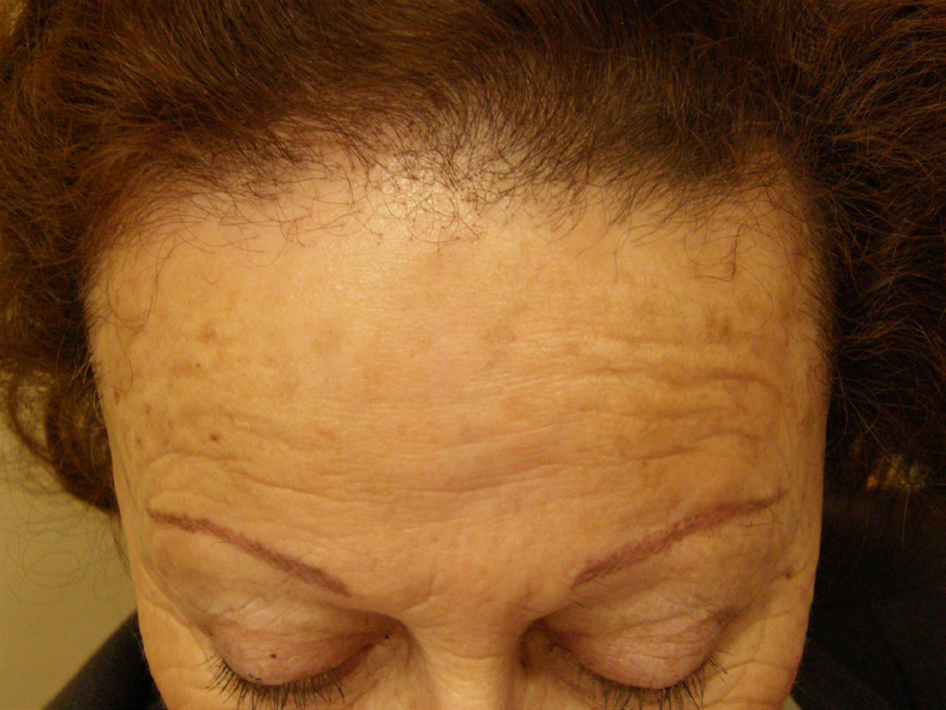
FFA: Scarring alopecia with recession of frontal hairline and alopecia of the eyebrows. Note peripilar papules

Frontal Fibrosing Alopecia (FFA) was first described in 1994 by Kossard ( 36 ) but its frequency is recently increasing worldwide. It is a lymphocytic cicatricial/scarring alopecia that is considered a variant of lichen planopilaris (LPP) (Figure 3). 

Patients are usually but not exclusively post-menopausal females (one review found the mean age to be 64 years) (37-39) who complain of slowly progressive recession of the frontal hairline.( 37, 38, 39) The temporal and parietal hairline can also be involved (38). It is also typical to see alopecia of the eyebrows and limbs (37-39). Sometimes a few scattered terminal hairs are seen in the band of recession. The new hairline shows absence of vellus and intermediate hairs, perifollicular erythema and scales around remaining terminal hairs (39).

The prevalence of this disease has been significantly increasing in the past 10 years (38) and even though no identifyable factors are to blame, this recent surge suggests that an enviromental factor can be implicated. The condition is slowly progressive and responds poorly to treatment.

## 8. Treatment of FPHL

The only medication approved for FPHL is 2% topical minoxidil. This section will however also discuss several other treatment alternatives that are possibly effective, but not approved. 

### 8.1. Minoxidil 2% Solution

This is a potassium channel opener and its mechanism of action is still unclear. It is believed that it enhances angiogenesis around the follicle by increasing the expression of vascular endothelial and hepatocytic growth factors, the latter being a hair growth promoter. It is also believed that there is activation of cyto protective prostaglandin synthase-1 (40). Minoxidil induces telogen hairs to enter the anagen phase, prolonging anagen duration (40). It increases hair count (40) and weight. Topical minoxidil solution should be applied only to the affected area of the scalp at the dosage of 1ml twice day (41) for a minimum period of 12 months before deciding about the efficacy. When effective, treatment should be continued indefinitely as with a chronic disease, otherwise, telogen effluvium can be triggered upon interruption. Patients should also be warned that in the first months of treatment a transient increase shed may occur. Treatment side effects are uncommon and include allergic or irritative contact dermatitis, which is more commonly related to the solution vehicle propylene glycol. This can be overcome with use of the 5% foam that does not contain this ingredient. A recent study showed that the 5% foam once a day was as effective as the 2% solution twice a day in female patients (42). Another possible side effect is hypertrichosis of the forehead or face, usually caused by accidental contamination or improper application. 

### 8.2. Anti-Androgens

Synthetic anti-androgens are used orally to to block androgen receptor binding. They include Cyproterone acetate (not available in the US), Spironolactone and Flutamide. Studies about efficacy of Cyproterone acetate are not uniform (43, 44). Probably it is more effective when there is evidence of biochemical hyperandrogenism (44). Spironolactone is a potassium sparing diuretic that acts by decreasing testosterone production in the adrenal gland and by blocking the androgen receptors in the target tissues (45). Although not approved to be used in FPHL, it has been shown to be effective in the treatment of hirsutism associated with polycystic ovarian syndrome and acne (46, 47). It is has been used off-label as an anti-androgen for FPHL at a dosage of 50 to 200 mg per day (48, 49) with better efficacy at 150 mg/day (50). One study showed equivalent efficacy in FPHL when Spironolactone was compared to Cyproterone acetate (49). Flutamide use is limited because it can cause severe liver toxicity. One study reported efficacy of Flutamide at a dosage rangeing from 62.5 to 250 mg day, without side effects at a low dosage; results were however only based on clinical examination (51). Another study found hepatic toxic side effects even with very low doses of Flutamide (52). Overall, there is not enough evidence based data to support the routine use of antiandrogens in FPHL.

### 8.3. Finasteride

Finasteride is a type two 5 alpha-reductase enzyme inhibitor which inhibits the convertion of testosterone into dihydrotestosterone (DHT) (40). Finasteride reduces hair loss and stimulates hair regrowth by increasing hair counts in men taking 1 mg daily (40). In women one controlled study with Finasteride 1 mg yielded no benefits on post-menopausal women (53). One uncontrolled study showed improvement in 62% of premenopausal women taking 2.5 mg of finasteride daily associated with an oral contraceptive containing drospirenone and ethinyl estradiol (54). Several case reports, case series and small trials confirmed improvement both in pre or postmenopausal women taking 2.5 to 5 mg of finasteride daily (55, 56). Response to treatment was not dependent on evidence of hyperandrogenism. Finasteride has a safe side-effect profile in men, but further controlled studies need to be conducted for extending the knowledge on its benefits and safety profile for women. Premenopausal women need to utilize safe contraception methods during treatment as the drug can cause feminization of the male fetus if taken throughout pregnancy. Another possible concern for this treatment is the slight rise of estrogen levels due to aromatase conversion of testosterone to estradiol. For this reason this treatment is not advisable in females with a family or personal history of breast cancer. 

### 8.4. Dutasteride 

Dutasteride is a type one and two 5 alpha-reductase enzyme inhibitor. It inhibits the conversion of testosterone to dihydrotestosterone. There are limited data about the use of this drug in women. There is one case report of good response after 6 months of treatment with 0.5mg/d of Dutasteride in a female patient who ceased to benefit from finasteride (57). Dr Camacho (58) reports improvement in 60% of 25 postmenopausal women with FPHL in the first year and 80% in the second year using very high dosages (0.25 mg/dL) of Dutasteride. Still according to the above mentioned author the association of Dutasteride 0.5 mg/d with Finasteride 2.5 mg/d was effective in a off-label study involving 14 postmenopausal women with FPHL and 5 premenopausal women with FPHL, hirsutism and nodulocystic acne (59).

A recent controlled study of 126 female patients evaluated the efficacy of locally injected Dutasteride in FPHL. Treatment was delivered by mesotherapy intradermally in the vertex. The solution contained 0.5 mg of Dutasteride, 20 mg of biotin, 200 mg of pyridoxin and 500 mg of D-panthenol in 2 ml. Injections were repeated weekly for 8 weeks than every 2 weeks, for 4 weeks and a last application at 16 weeks. Photographic improvement occured in 62.8% of treated patients at the 18th week (60).

### 8.5. Estrogens

Estrogens have an uncertain role in human hair growth. The hair follicle has different estrogen receptors: alpha and beta. The beta receptor is the most common one present in the scalp and in general it suppresses cellular function in the hair follicle. Studies in vitro are inconclusive and they show that estrogens may have opposite effects in male scalp hair, where they induce stimulation, versus female scalp hair where they inhibit hair elongation (61, 62). Precursor androgens can be transformed into estrogens in the hair follicle (due to the presence of aromatase and 17-hydroxy steroid dehydrogenase in loco) (63) and estrogens may affect the amount of DHT by affecting the function of the 5-alpha reductase enzyme (64). It has been suggested that a low estrogens to androgens ratio could favour the development of the disease in the genetically susceptible individuals (65). Controlled studies regarding efficacy of topical estrogens for hair loss show controversial results (66-68).

### 8.6. Lasers and Light Treatments 

Lasers and light treatments are monochromatic lights that utilize wavelengths between 600 to 1,400nm, in the red/infrared spectrum (69). There is some evidence that light treatments can stimulate hair growth and the mechanism by which this happens is uncertain (70). The light treatment effects might be attributed to the absorption of red/infrared light by the skin which than is absorbed by the cellular respiratory chain (71). The Lasercomb Hair MaxR is a portable laser device that uses a wavelenght of 655nm widely marketed for patients as a hair regrowth device. There is one controlled study in males showing efficacy of this technology (72) but there are no published studies in women. 

### 8.7. Prostaglandin Analogs

latanoprost and bimatoprost were initially developed for eye glaucoma and one side effect noticed was the growth of eye lashes. There is one study in men showing that lanatoprost 0.1% increased scalp hair density compared to baseline and placebo (73) but the study included only 16 male patients and the medication was applied to a very small area of the scalp. Different classes of prostaglandins seem to have opposite actions in the hair follicle.

### 8.8. Hair Transplantation

When the loss of hair has been stabilized in patients over 25 years old, hair transplantantion is an alternative. The gold standard technique is the follicular unit transplantation, because of a better outcome in terms of natural architecture and final aspect (74). The hair follicles are implanted individually following the patients natural hair line. It is a multi-step procedure that should be performed by an experienced surgical team. The results will depend on sufficient donor area, number of transplanted hairs, the quality of the hair harvested and the recipient area. The most common problems encountered in hair transplantion in women are related to insufficient hair donor areas, the need for magnification to insert the grafts between the existing hair follicles present in the recipient area and temporary worsening of global aspect after the transplant. 

## 9. General Care 

Patients should maintain a healthy and varied diet to guarantee adequate iron, vitamins and protein intake. They should avoid local treatments and manipulation of the scalp that could cause hair breakage (such as straightening, perming and hair extensions), which can mimic hair loss. If possible, avoid medications prone to cause hair shedding and other factors that could negatively impact the hair growth, such as smoking and sun exposure of the scalp (75, 76).

## 10. Conclusions

FPHL is a common condition that cause considerable distress to patients. Recent data indicate that FPHL is not just a cosmetic problem but it is significantly associated with metabolic syndrome and its medical complications. Prompt diagnosis, evaluation of comorbidities and treatment are important and management of this condition often involves a multidisciplinary approach.
